# Monopolistic Control of Medical Literature

**Published:** 1897-05

**Authors:** 


### Correspondence.

### MONOPOLISTIC CONTROL OF MEDICAL LITERATURE.

*To the Editor:*

Sir—I regret exceedingly again to trespass upon your courtesy, but it seems impossible not to do so, if your readers are to have even a partially correct understanding of the facts in controversy. The replies and statements of Messrs. Wood & Co. with wearisome reiteration emphasise the assertion that I personally " injected myself" into the controversy and spoiled its assured settlement " to the satisfaction of all parties," and that the refusal of Messrs. Wood & Co. to allow the use of quotations and cuts from their periodicals was after extended controversy, and after their long-suffering had been worn out by eighteen months' exhaustion from the importunity of Mr. Saunders in asking for an excessive number of electros and " wholesale use of our facilities" (sic !), etc., etc. That this is utterly untrue the following letter will make plain. Note that the date is over one year ago :

William Wood & Company,
Medical Publishers.

W. B. Saunders, New York, March 11, 1896.
925 Walnut street, Philadelphia, Pa.

Dear Sir—Your form letter addressed to the editor of the *American Journal of Obstetrics*, requesting two copies of the *Journal* to be sent you for use in the publication of your Year Book, has been handed to us for reply. Neither the *Journal of Obstetrics* nor the *Medical Record*, both published by us, consider that they are under any obligations whatever for such quotations as proposed by you, but the contrary. We are obliged, from the course pursued by various parties in the past, to say to you that both of these journals are copyrighted and everything in them protected by law. Quotations from either of them are not permitted for such use as proposed by you.

Respectfully yours,
WM. WOOD & CO.

By necessary inference this letter settles the inaccuracy of many other statements and insinuations in the published letters of Messrs. Wood & Co., that are too wearying to detail, and especially the following astonishing sentence : " We never refused any request of Mr. Saunders to abstract from our publications, nor, with one exception a long time ago, have we ever declined to allow him the use of cuts."

To the charge also made that I do not give "the slightest acknowledgment of the benefits" received to authors and periodicals quoted, I would add that *by actual count* I find that in the three books under discussion, there are in all 437 credits to the *Medical Record* and the *American Journal of Obstetrics*. If there is any omission it is an oversight for which I am sorry, as it is at once a matter of pride, self-interest and duty to give proper journal-references and credits for all excerpts, abstracts and illustrations. He would be a strange editor who failed in this. To be charged with making "intentionally misleading statements" (anglicé, lies) by one who lives in such a very glazed house is illustrative of the humors and psychologic puzzles of a controversy that has doubtless over-much tired your indulgent good-will and that of others. Cordially yours,

GEO. M. GOULD,

April 7, 1897. 119 S. 17th St., Philadelphia.

